# Antibody deficiency in patients with frequent exacerbations of Chronic Obstructive Pulmonary Disease (COPD)

**DOI:** 10.1371/journal.pone.0172437

**Published:** 2017-02-17

**Authors:** Brian N. McCullagh, Alejandro P. Comellas, Zuhair K. Ballas, John D. Newell, M. Bridget Zimmerman, Antoine E. Azar

**Affiliations:** 1 Division of Pulmonary and Critical Care, Department of Internal Medicine, University of Iowa Hospitals and Clinics, Iowa City, Iowa, United States of America; 2 Division of Immunology, Department of Internal Medicine, University of Iowa Hospitals and Clinics, Iowa City, Iowa, United States of America; 3 Departments of Radiology and Biomedical Engineering, University of Iowa, Iowa City, Iowa, United States of America; 4 Department of Biostatistics, College of Public Health, University of Iowa, Iowa City, Iowa, United States of America; 5 Division of Allergy and Clinical Immunology, Department of Internal Medicine, the Johns Hopkins University School of Medicine, Baltimore, Maryland, United States of America; National and Kapodistrian University of Athens, SWITZERLAND

## Abstract

Chronic Obstructive Pulmonary Disease is the third leading cause of death in the US, and is associated with periodic exacerbations, which account for the largest proportion of health care utilization, and lead to significant morbidity, mortality, and worsening lung function. A subset of patients with COPD have frequent exacerbations, occurring 2 or more times per year. Despite many interventions to reduce COPD exacerbations, there is a significant lack of knowledge in regards to their mechanisms and predisposing factors. We describe here an important observation that defines antibody deficiency as a potential risk factor for frequent COPD exacerbations. We report a case series of patients who have frequent COPD exacerbations, and who were found to have an underlying primary antibody deficiency syndrome. We also report on the outcome of COPD exacerbations following treatment in a subset with of these patients with antibody deficiency. We identified patients with COPD who had 2 or more moderate to severe exacerbations per year; immune evaluation including serum immunoglobulin levels and pneumococcal IgG titers was performed. Patients diagnosed with an antibody deficiency syndrome were treated with either immunoglobulin replacement therapy or prophylactic antibiotics, and their COPD exacerbations were monitored over time. A total of 42 patients were identified who had 2 or more moderate to severe COPD exacerbations per year. Twenty-nine patients had an underlying antibody deficiency syndrome: common variable immunodeficiency (8), specific antibody deficiency (20), and selective IgA deficiency (1). Twenty-two patients had a follow-up for at least 1 year after treatment of their antibody deficiency, which resulted in a significant reduction of COPD exacerbations, courses of oral corticosteroid use and cumulative annual dose of oral corticosteroid use, rescue antibiotic use, and hospitalizations for COPD exacerbations. This case series identifies antibody deficiency as a potentially treatable risk factor for frequent COPD exacerbations; testing for antibody deficiency should be considered in difficult to manage frequently exacerbating COPD patients. Further prospective studies are warranted to further test this hypothesis.

## Introduction

Chronic Obstructive Pulmonary Disease (COPD) is the third leading cause of death in the US since 2008, and the primary contributor to mortality caused by chronic lower respiratory diseases [1]. The World Health Organization predicts that COPD will become the third leading cause of death worldwide by 2030. In the US, the estimated direct costs of COPD are $29.5 billion and indirect costs $20.4 billion annually [2].

COPD is associated with periodic respiratory exacerbations that manifest as worsening lung function and increased dyspnea, cough and sputum production. These acute exacerbations of COPD (AECOPD) are more frequent with increased disease severity and continuation of tobacco use, occurring 1–3 times per year [3]. AECOPD accounts for the largest proportion of the total COPD burden on health care utilization [4]. These exacerbations are clearly an important contributor to quality of life, morbidity and mortality of patients with COPD, and frequent exacerbations are linked to a more rapid decline in lung function [5]. A subset of patients with COPD have frequent exacerbations, ≥2/year. Interestingly, the best-known predictor of AECOPD in this “frequent exacerbation” phenotype is a prior history of exacerbation [3]. Strategies to prevent exacerbations currently focus on several aspects including: i) reducing exposure to offending agents such as tobacco smoke; ii) decreasing lung inflammation and optimizing airway bronchodilation; and iii) preventing infection using influenza and pneumococcal vaccines [2]. Despite all these interventions, and the well-documented magnitude of this problem, there remains a significant lack of knowledge in regards to the mechanisms of AECOPD, especially in frequent exacerbators.

It is estimated that 70–80% of AECOPD are due to respiratory infections; 50% or more are considered bacterial in nature, mostly caused by encapsulated organisms [6] (e.g. *Streptococcus pneumoniae*). Several mechanisms are involved in lung immunity to defend against respiratory tract infections. These include innate, cellular and humoral immunity. Humoral immunity is the main defense mechanism against encapsulated organisms, and plays a key role in preventing respiratory infections. Defects in humoral immunity are associated with a significantly increased risk of respiratory tract infections, in particular sino-pulmonary infections (rhinosinusitis, otitis, bronchitis, pneumonia), caused by encapsulated bacteria, organisms implicated in AECOPD. Therefore, we hypothesized that patients with frequent AECOPD may have a defect in humoral immunity that predisposes them to recurrent respiratory infections and therefore AECOPD.

Humoral immune defects are a spectrum of diseases that represent the most common primary immunodeficiency disorders. The adult antibody deficiency syndromes include Common Variable Immunodeficiency (CVID), selective IgA deficiency (SIgAD), and specific (functional) antibody deficiency (SAD). The prevalence of antibody deficiencies ranges from 1:500 in SIgAD to about 1:25,000 in CVID. About 32% of patients with CVID develop pneumonia, and about 23% develop bronchiectasis [6]; respiratory disease is the most common cause of death in patients with CVID [7]. Immunoglobulin (IG) replacement therapy is the mainstay therapy to treat CVID, and it reduces the frequency and severity of respiratory infections and improves quality of life [8].

Patients with antibody deficiency syndromes are, therefore, at a significantly increased risk of infection with a similar spectrum of organisms responsible for AECOPD. Despite this, there are almost no reports linking AECOPD with the antibody deficiency syndromes. Only recently, Cowan et al reported a retrospective analysis of 14 cases of COPD patients who were on IG treatment [9]. They described that IG treatment decreased the frequency of moderate and severe recurrent AECOPD. Although a subset of patients in this report had low serum IgG, they did not undergo an evaluation or diagnosis of a humoral immunodeficiency prior to treatment with IG therapy. Here we hypothesize that antibody deficiency is more prevalent than expected in patients with frequent AECOPD compared to that of the general population, and represents an important risk factor for frequent AECOPD.

The diagnosis of the antibody deficiency syndromes can be challenging. Diagnosis relies on measurement of serum IG levels. However, functional specific antibody deficiency is frequently missed with these screening labs, since the IG levels are usually normal in these patients. It is imperative to obtain a qualitative, as well as a quantitative, assessment of antibody function when a defect in humoral immunity is suspected. One of the primary means for assessment of antibody function relies on documenting an impaired specific antibody response to polysaccharide vaccines; assessing IgG antibody response to the pneumococcal polysaccharide vaccine (Pneumovax, PPV23) is the most commonly used and best-studied test [10–12].

We present a single center experience identifying a case series of patients with frequent moderate and severe AECOPD who were diagnosed with humoral immunodeficiency. Furthermore we report that in our cohort of patients with concomitant COPD and immune deficiency treated with either IG therapy or prophylactic antibiotics, a significant reduction in AECOPD frequency, hospitalizations, use of rescue antibiotics and systemic corticosteroids was observed.

## Materials and methods

### Patient selection

The institutional review board of the University of Iowa Hospitals and Clinics approved this study. The IRB waived the need for written informed consent.

Our study had two arms. Firstly we describe a case series of COPD patients suffering from frequent exacerbations who had a concomitant diagnosis of immunodeficiency. Secondly we identify a subset of patients for whom we had data pre and post therapeutic targeting of their immunodeficiency to determine if this had a significant effect on their exacerbation history.

We identified patients with COPD seen in the authors’ Pulmonary Clinic between January of 2012 and December of 2014 with 2 or more exacerbations per year and were referred to the Allergy/Immunology Clinic for immune evaluation. All AECOPD were classified as either moderate or severe requiring the use of prednisone and/or antibiotics as an outpatient (moderate) or inpatient (severe). All patients had blood tests done to check CBC with differential, serum Immunoglobulins (IgG, IgA, IgM, IgE), as well as antibody IgG response to the pneumococcal vaccine (PPV23). Additional immune evaluation was performed as directed by their clinical history. Patients on oral corticosteroids had their serum IGs repeated when off steroids; for patients where oral corticosteroid use could not be discontinued, serum IGs were repeated on the lowest tolerated dose of oral corticosteroids. Informed consent was not obtained from patients who were reported in this study since the data was analyzed anonymously.

Additionally we further identified patients with a concomitant diagnosis of COPD and immune deficiency by performing a retrospective chart search. This search consisted of all patients with COPD, who also carried the diagnosis of immunodeficiency (searched for immunodeficiency, CVID, antibody deficiency, IgA deficiency), or had any of IgG, IgA, IgM or pneumococcal IgG titers ordered between January of 2010 and Dec 2014. We recorded the details of these patients who had at least 2 annual moderate or severe AECOPD.

Patients found not to have documented COPD on PFTs, frequent moderate or severe COPD exacerbations, or who did not meet diagnostic criteria for immunodeficiency or had secondary immunodeficiency (e.g malignancy, medications), were excluded.

In an effort to determine if treatment directed at patients immunodeficiency might improve COPD exacerbation frequency we recorded the following parameters at least one and up to 10 years before and after diagnosis and treatment initiation: number of COPD exacerbations per year, number of hospitalizations, ICU admissions, annual courses of antibiotics, annual courses of oral corticosteroids for COPD exacerbations, and the cumulative annual dose of corticosteroids used for COPD.

### Diagnosis of COPD

In subjects with a smoking history the diagnosis of COPD was confirmed by spirometry (FEV1/FVC<0.70). GOLD classification based on post bronchodilator FEV1 was used to stratify severity of disease. GOLD 1: mild, FEV1 ≥8 0% predicted; GOLD 2: Moderate, 50% ≤ FEV1 <80% predicted; GOLD 3: Severe, 30% ≤ FEV1 < 50% predicted; and GOLD 4: Very severe, FEV1<30% predicted. In the absence of PFTs a CT chest with evidence of emphysema was accepted.

### Serum immunoglobulin measurement

Serum samples were assayed for total IgG, IgA, IgM, and IgE by nephelometry on Roche Diagnostics (Indianapolis, IN) cobas c701 analyzers in the core clinical chemistry laboratory at the University of Iowa Hospital and Clinics (UIHC).

### Pneumococcal IgG titers measurement

IgG titers to *Streptococcus pneumoniae* serotypes were sent to ARUP laboratories and processed by quantitative multiplex bead assay. Pre and post vaccination samples were used to determine humoral immune response to the vaccine. Subjects who had received the pneumococcal vaccine with the past 12 months had random titers checked. In our institution the 14 serotype vaccine was changed to the 23 serotype vaccine during the course of our analysis.

### Immunophenotyping

Lymphocyte immunophenotyping was performed using flow cytometry at the University of Iowa Hospital core flow cytometry lab.

### Diagnosis of immunodeficiency

Patients were diagnosed with a primary antibody deficiency syndrome per published guidelines, as evidenced by a reduction in serum IG levels below standardized reference ranges and/or inadequate response to pneumococcal vaccination. The diagnosis of common variable immunodeficiency (CVID) was based on a serum IgG level that is more than 2 SDs below the mean, with a low serum IgA and/or IgM, and a deficient antibody response to vaccines [11].

The diagnosis of specific antibody deficiency was made based on a deficient IgG antibody response to PPV23, with normal serum IG levels. Antibody response to PPV23 was interpreted based on the most recent published consensus parameters [12] (Table 1). The diagnosis of selective IgA deficiency was defined as a serum IgA level that is less than 7 mg/dL (lowest detectable laboratory limit), with normal serum IgG and IgM [11].

**Table 1:** PPV23-deficient response phenotypes.

Phenotype	PPV23 response
Severe	≤2 protective titers (≥1.3 mcg/mL)
Moderate	<70% of serotypes are protective (≥1.3 mcg/mL)
Mild	Failure to generate protective titers to multiple serotypes or failure of a 2-foldincrease in 70% of serotypes^a^

^a^ 2-Fold increases assume a prevaccination titer of less than 4 mcg/mL

Adapted from: Orange et al. *J Allergy Clin Immunol*. 2012; 130(3 Suppl):S1-24.

### Treatment protocol for immunodeficiency

Patients diagnosed with CVID were recommended for treatment with intravenous (IVIG) or subcutaneous (SCIG) immunoglobulin replacement therapy. IVIG was administered every 3 or 4 weeks, and SCIG was administered every 1 or 2 weeks. The total dose of IVIG or SCIG ranged from 300 to 600 mg per Kg per 4-week period. Patients diagnosed with specific antibody deficiency were recommended treatment with prophylactic antibiotics; the choice of prophylactic antibiotic was either alternating every 2-weeks Trimethoprim/Sulfamethoxazole (1 DS tablet twice daily) and doxycycline (100 mg twice daily), or alternate day azithromycin 250 or 500mg orally on Monday/Wednesday/Friday. Patients were treated with these antibiotics for the duration of their care. Patients with specific antibody deficiency who continued to have recurrent infections despite prophylactic antibiotics were started on IG replacement therapy. Patients with selective IgA deficiency were treated with prophylactic antibiotics only.

### Chest computed tomography

The most recent clinical chest CT scan from all patients were read by an expert radiologist (JN) in a blinded fashion. Comparison was made between patients who required IG replacement therapy versus those who did not. The radiological characteristics that were recorded included presence of emphysema, bronchiectasis, ground glass opacities and centrilobular nodules. Emphysema was classified as Centriacinar, Panacinar, Distacinar, and Mixed. Bronchiectasis was classified as cylindrical, varicoid, and cystic. These findings were determined in each lung lobe. Analysis of the results was performed by Chi-square using GraphPad Prism 6.0.

### Statistics

Descriptive statistics for the general patient characteristics were computed for all patients, and by type of therapy. These included frequency counts and percentages for categorical variables, and mean and standard deviation (SD), or median and interquartile range (IQR) for continuous variables. Patient characteristics and CT scan findings were compared between the therapy groups using Pearson chi-square or Fisher’s exact test for the categorical variables, and two-sample t-test or Wilcoxon rank-sum test for the continuous variables. Changes in pulmonary function test measures before and after treatment of antibody deficiency were assessed using the paired t-test. Improvement in patient outcomes before and after treatment of antibody deficiency were tested using Wilcoxon signed-rank test for continuous variables (number of exacerbations, hospitalizations, prednisone dose, courses of rescue antibiotics), and McNemar’s test for categorical variables (ICU admission, oxygen use). All statistical analyses were performed using SAS (version 9.4).

## Results

We identified 42 patients with ≥2 AECOPD per year and had an immune workup performed for evaluation of antibody deficiency between January 2012 and December 2014. Twenty-eight patients were identified through the pulmonary clinic as patients on maximal inhaled therapy for COPD (LABA, LAMA and inhaled corticosteroids) yet were still suffering from frequent AECOPD and therefore had been referred to Allergy & Immunology for further work up. The remaining 14 patients were subsequently identified using a retrospective chart search.

Of the 42 patients, 7 were excluded, as there was no documentation of obstructive airways disease on PFTs and therefore the diagnosis of COPD could not be confirmed upon chart review. Three patients without PFT evidence for obstructive airways disease had emphysema on CT and were included. Four patients did not have a confirmed antibody deficiency given that in spite of documented hypogammaglobulinemia they had an adequate IgG response to PPV23 vaccination. Two patients had hypogammaglobulinemia but did not have functional antibody assessment done at the time of writing and were excluded.

**Fig 1:**
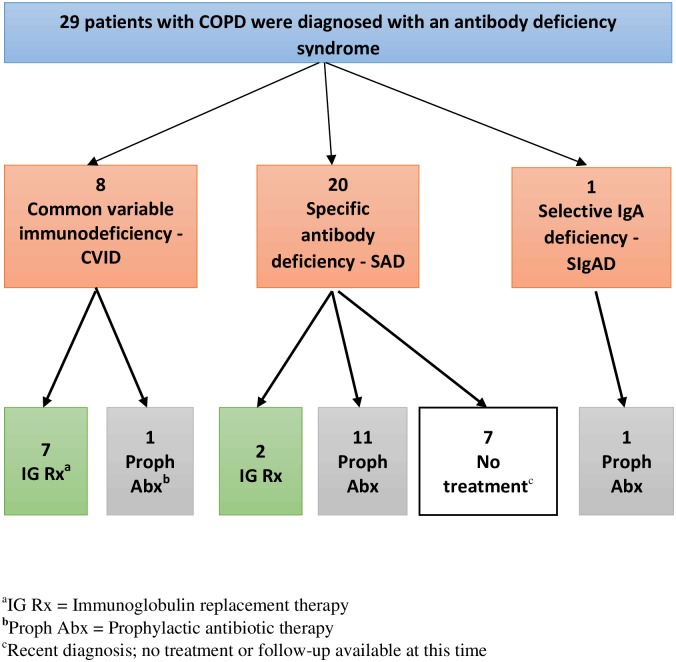
Classification of patients with COPD and antibody deficiency. 42 patients were identified initially and 1 excluded. Twenty-nine patients were confirmed to have a concomitant diagnosis of COPD with frequent exacerbations, and an antibody deficiency syndrome (CVID, SAD, SIgAD). Seven of 8 patients with CVID were treated with IG replacement, 1 patient declined and was treated with prophylactic antibiotics. Eleven of 20 patients with SAD were treated with prophylactic antibiotics. Those who failed treatment with prophylactic antibiotics (n = 2) were treated with IG replacement. Seven patients had not received treatment by the time of writing. 1 patient with SIgAD was treated with prophylactic antibiotics.

We describe a total of 29 patients with poorly controlled COPD as evidenced by ≥2 moderate to severe AECOPD per year and with documented evidence of an antibody deficiency syndrome. Among this group 8 had CVID, 20 had specific antibody deficiency and 1 had selective IgA deficiency (Fig 1).

Of 8 patients with CVID, 7 were treated with IG replacement while 1 declined IG replacement and received prophylactic antibiotics. Of 20 patients with antibody deficiency, 11 were treated with prophylactic antibiotics, 2 with IG replacement because of recurrent infections and COPD exacerbations despite prophylactic antibiotics, and the remaining 7 were diagnosed with specific antibody deficiency but had yet to be commenced on treatment. The one patient with selective IgA deficiency was treated with prophylactic antibiotics (Fig 1).

There were marginally more females (n = 16) than males (n = 13) in the immunodeficiency population with a mean age at diagnosis of 65.8 years (Table 2). Average smoking history in pack years was 45; smoking history ranged from 15 to 75 pack-years. A total of 8 patients were current smokers. Although smoking pack-years were significantly higher in the subset of patients treated with IG replacement, the baseline FEV1 was not significantly different between the 2 groups. Three patients in the IG replacement group were current smokers, while 5 patients in the prophylactic antibiotic group were current smokers. Mean FEV1% was 49.3 (13–101) giving a mean GOLD classification of 3 or severe obstructive airways disease.

**Table 2:** General characteristics of patients with frequent COPD exacerbations and concomitant primary antibody deficiency syndrome (n = 29). The only statistical difference between patients treated with prophylactic antibiotics versus immunoglobulin replacement therapy was in the smoking history and serum IgG levels.

Variable	All patients (n = 29)	Prophylactic Antibiotic Therapy (n = 13)	Immunoglobulin Replacement Therapy(n = 9)	p-value
Gender (Male)	13 (45%)	6 (46%)	5 (56%)	1.0
Age, mean (SD)	65.8 (7.4)	64.9 (10.1)	66.9 (7.1)	0.622
Age at diagnosis, mean (SD)	61.8 (6.5)	60.8 (7.2)	62.4 (5.8)	0.571
Smoke pack-year, median [IQR]	45 [30–50]	30 [15–45]	50 [45–75]	**0.028**
Current smokers	8	5	3	**NS**
FEV1, mean (SD)	49.3 (18.9)	53.4 (22.3)	42.7 (16.2)	0.28
Quantitative immunoglobulins, median—mg/dL [IQR]				
IgG (694–1618)	561 [393–650]	602 [556–734]	371 [285–563]	**0.038**
IgA (68–378)	106 [47–181]	89 [63–166]	140 [28–196]	1.0
IgM (60–263)	67 [40–116]	79 [50–116]	40 [31–63]	0.142
Quantitative immunoglobulins in normal range—mg/dL				
IgG (694–1618)	6 (24%)	4 (31%)	2 (22%)	0.658
IgA (68–378)	16 (64%)	8 (62%)	5 (56%)	0.779
IgM (60–263)	14 (56%)	8 (62%)	3 (33%)	0.193

IQR = Interquartile range (25^th^-75^th^ percentile)

### Reduced immunoglobulin levels and pneumococcal antibody response in patients with AECOPD

The percentage and median levels of baseline IgG, IgA, and IgM immunoglobulin levels are shown in Table 2. Nine patients were taking oral corticosteroids at the time of sampling for immunoglobulin levels, 5 had repeat testing off steroid. The remaining 4 patients had repeat testing on the lowest dose of oral steroid tolerable (20 mg of prednisone daily in 2 patients, 10 mg of prednisone daily in 2 patients). There was a statistically significant lower IgG level (p = 0.038) in patients who received immunoglobulin replacement therapy compared to patients who were treated with prophylactic antibiotics (371 vs 602 mg/dL respectively).

All patients had pneumococcal antibody assessment done prior to the diagnosis of antibody deficiency. Of the 29 patients who were diagnosed with an antibody deficiency syndrome, 18 had pre/post PPV23 immunization titers done, and 11 patients had random titers done, since they had received the pneumococcal vaccine within the past 12 months. low cytometry did not show abnormalities in the numbers of T and B lymphocytes.

### Pulmonary function

Pulmonary function testing (PFTs) was available in 26 of 29 subjects with combined COPD and immune deficiency. The 3 individuals without available PFTS had radiological evidence of emphysema. PFTs demonstrated obstructive lung disease with a mean FEV1 of 49.3% consistent with GOLD classification stage 3 i.e severe COPD.

Patients were classified according to GOLD criteria follows: GOLD stage 1 (n = 1); GOLD stage 2 (n = 11); GOLD stage 3 (n = 11), and GOLD stage 4 (n = 3) (Table 3). Only 9 patients had PFT testing done before and after initiation of treatment for immune deficiency; there was no change in pulmonary function following treatment (Table 4). There was no significant difference in FEV1 between patients who were treated with prophylactic antibiotics or immunoglobulin replacement therapy (Table 2).

**Table 3:** GOLD classification in patients with COPD, including those who were treated with antibiotics versus immunoglobulin replacement.

Variable, mean (SD)	Before Treatment	After Treatment	Change	95% CI	Paired t-test p-value
FVC	72.4 (22.6)	79.3 (20.9)	6.9 (23.6)	-11.3, 25.0	0.407
FEV1	45.2 (25.7)	47.8 (19.9)	2.6 (17.5)	-10.9, 16.0	0.673
FEV1/FVC	47.4 (13.6)	50.7 (19.9)	3.2 (11.1)	-5.3, 11.8	0.410

**Table 4:** Pulmonary function tests before and after treatment of antibody deficiency in patients with COPD, in the subset of patients where this was performed (n = 9). There was no significant change following treatment of antibody deficiency.

	Total number of patients with PFTs (n = 26)	Prophylactic Antibiotic Therapy (n = 12)	Immunoglobulin Replacement Therapy (n = 7)
GOLD1	1 (3.8)	1 (8.3)	0
GOLD2	11 (42.3)	5 (41.7)	3 (42.8)
GOLD3	11 (42.3)	5 (41.7)	2 (28.6)
GOLD4	3 (11.5)	1 (8.3)	2 (28.6)

### CT scan analysis

Out of all the patients with COPD and immunodeficiency, all 8 patients who received IG replacement had lung CT scans, while only 18 of the non-IG patients had lung CT scans performed. Emphysema was more prevalent in all lung lobes in the IG group compared to the non-IG (p <0.01). Also, the most common type of emphysema in the IG group was centriacinar in all lobes except for the right upper lobe, whereas distal acinar was more prevalent in the IG group. Bronchiectasis was present in all lobes. Specifically, the group that did not receive IG therapy had more bronchiectasis in right upper, right middle, right lower and left lower lobes compared to the IG therapy group (p = 0.0254) (Fig 1). However the majority, approximately 90%, were cylindrical, with very few varicoid or cystic, the latter considered more severe bronchiectasis. Interestingly, the non-IG group had a significantly higher percentage of bronchiectasis compared to the IG group. Ground glass opacities were not different between groups. Finally, the IG group had more centrilobular nodules, predominantly in both upper lobes. These results are within the expected prevalence of CT findings in COPD patients [13].

### Reduction in COPD exacerbations with treatment of antibody deficiency

At least 1-year follow up data post initiation of targeted therapy for immune deficiency was available for 22 patients. With the introduction of targeted therapy the annual rate of AECOPD fell from 4 (3–6) to 1 (0–2) (p<0.0001). Average annual courses of oral steroid reduced from 4 (0–12) to 0 (0–1) (p = 0.004) with the overall cumulative annual dose of steroid (mg) declining from 930 (0–3075) to 0 (0–1) (p = 0.004). Average number of courses of rescue antibiotics declined from 6 (4–12) to 0 (0–1) (p<0.0001). Hospitalizations for AECOPD fell from 1 (0–2) to 0 (p = 0.037). Two ICU admissions were recorded prior to initiation of targeted therapy with none recorded following treatment (Table 5).

**Table 5:** Data on exacerbations, hospitalizations, prednisone use, and rescue antibiotic use, before and after treatment of antibody deficiency, in patients diagnosed with COPD and concomitant antibody deficiency syndrome.

Variable	n	Before	After	Change	p-value^a^
Exacerbations/year, median [IQR]^b^	18	4 [3–6]	1 [0–2]	-3.5 [-5 to -2]	<0.0001
Hospitalizations for AECOPD/year	17	1 [0–2]	0 [0–0]	-1 [-1 to 0]	0.037
ICU admissions	18	2 (11%)	0 (0%)		0.500
Prednisone cumulative annual dose	12	930 [0–3075]	0 [0–40]	-310 [-1990 to 0]	0.031
Average courses of prednisone/year	15	4 [0–12]	0 [0–1]	-3 [-11 to 0]	0.004
Average courses of rescue antibiotics/year	19	6 [4–12]	0 [0–1]	-6 [-10 to -3]	<0.0001
Oxygen use	20	7 (35%)	9 (45%)		0.157

^a^ p-value from Wilcoxon signed-rank test, except ICU admissions and Oxygen use which was from McNemar’s test

^b^ IQR = Interquartile range (25th-75th percentile)

In patients treated with prophylactic antibiotics only, there was a reduction in the number of exacerbations (p = 0.004) and courses of rescue antibiotics (p = 0.002). In patients treated with IG replacement, there was a reduction in exacerbations (p = 0.016), courses of prednisone (p = 0.031), and courses of rescue antibiotics (p = 0.016); numbers were too small to see a difference in annual rates of hospital admission for AECOPD pre-treatment 1.5 (1–3) and post-treatment 0 (0–1.5) (p = 0.25).

In the subgroup of patients receiving IVIG (n = 8) we observed a reduction in annual courses of steroid from 12 (4.5–12) to 0.5 (0–1.5) (p = 0.031) and annual courses of rescue antibiotic from 9 (5.5–12) to 0 (0–1.5) (p = 0.016). Annual acute exacerbations in this group fell from 4 (3–5.5) to 0.5 (0–1.5) (p = 0.016).

## Discussion/Conclusions

COPD is now accepted to be a heterogeneous condition with many varying phenotypes [14, 15]. Defining these phenotypes will enable a more tailored, patient centered treatment approach. The frequent exacerbator phenotype is of particular importance because of the more rapid decline in lung function and increased utilization of health care resources among this group [3]. This case series identifies a subset of patients with frequent AECOPD who have an underlying, clinically significant, humoral immunodeficiency; to our knowledge, this has not been previously described. This suggests that antibody deficiency is much more common in patients with frequent AECOPD as compared to the historical norm. It is estimated that the prevalence of CVID in the general population is ~ 1:25,000; the prevalence of SAD is less well defined, but is probably at least as common as CVID [16–19]. Selective IgA deficiency has a prevalence of about 1:500, but the majority of patients with selective IgA deficiency are asymptomatic. Based on this series and our experience, it seems that the prevalence of CVID and SAD in patients who have frequent AECOPD despite being on maximal medical therapy for COPD, is much higher than the general population. This would not be unexpected given that patients with humoral immunodeficiencies develop respiratory infections with similar organisms that are responsible for COPD exacerbations. We therefore demonstrate a sub-phenotype of COPD patients that frequently exacerbate and who have concomitant humoral immune deficiency.

Immune deficiency has long been known to be associated with bronchiectasis. Almost all types of immunodeficiency syndromes can theoretically lead or contribute to bronchiectasis by increasing the risk of chronic or abnormally frequent and serious sinopulmonary infections [20, 21]. Humoral immunodeficiencies have been the most studied, and bronchiectasis is one of the most common complications in patients with antibody deficiency [22–28]. Prevalence of bronchiectasis has varied from 23% to 65% in patients with common variable immunodeficiency [6, 29]. It is conceivable that in the setting of obstructive airways disease, antibody deficiency and frequent pulmonary exacerbations, we may have been solely identifying bronchiectatic patients. We therefore examined all available CT scans from our patient population with a blinded radiologist to determine the extent of airways inflammation, emphysema and bronchiectasis. Our finding of mild bronchiectasis in this group is further evidence to support our principle hypothesis that these exacerbations were mainly due to COPD and not bronchiectatic exacerbations. Also, in our group of patients there was no improvement of PFTs upon the initiation of treatment for immunodeficiency. Quinti et al [8] reported that upon initiation of treatment for primary antibody deficiency, patients’ lung function had some improvement. Our result suggests that lung disease in these patients is primarily due to COPD, a fixed obstructive defect, and not due to primary antibody deficiency or reversible airway disease.

The second arm of our study proposes that treatment of the underlying antibody deficiency disorder (with either immunoglobulin replacement or prophylactic antibiotics) can result in a clinically meaningful improvement in number of AECOPD, number of hospitalizations for AECOPD, number of prednisone courses per year, cumulative annual prednisone dose, and number of courses of rescue antibiotics. It could be argued that our population had incentive to quit smoking following a diagnosis of immunodeficiency and this might contribute to the reduction in rate of exacerbation. Eight of 22 patients were current smokers in our treated population for whom we observed similar improvements in exacerbation rate and rescue medication usage compared to ex-smokers (n = 14). While the numbers are too small to draw any concrete conclusions we can say that smoking status is unlikely to have been a confounder in our group.

As expected, the lung function and oxygen use did not change with treatment. Recently, a clinical trial using daily azithromycin also demonstrated a reduction in AECOPD frequency [30]. While the authors of this study consider the anti-inflammatory effect of azithromycin, our results raise the possibility that the beneficial effects are also from treating patients with concomitant humoral immunodeficiency. Despite the evidence that use of continuous prophylactic antibiotics results in a clinically significant benefit in reducing AECOPD [31], it is unclear how to select the patients that will benefit from this intervention [32]. This study therefore highlights some additional diagnostic avenues for these difficult to treat patients.

There are limitations to this study principally that it is a retrospective observational single center study, and there are no matched controls. AECOPD events are difficult to differentiate from primary bronchiectatic exacerbations. However, the presence of mild bronchiectasis on CT and the lack of PFT improvement after initiation of IG or rotating antibiotics supports that these events were mainly AECOPD. Our center is also a tertiary referral center for both difficult to treat COPD and immunodeficiency disorders, and so a referral bias may over-represent the coexistence of these pathologies. This is a highly selective group of patients who were found to have an unusual number of exacerbations as recognized by experienced pulmonary physicians.

For future directions, this important finding requires to be tested prospectively in a large cohort of well-characterized COPD patients, starting with the assessment of the prevalence of antibody deficiency syndromes in patients known to have frequent AECOPD, compared to a control group of patients with COPD who do not have frequent exacerbations and monitor their clinical response to antibody deficiency treatment.

In spite of its limitations, this study raises the important awareness of the coexistence of these two conditions, thereby reporting for the first time a potentially important treatable cause of frequent exacerbations in COPD patients. In addition, our results are consistent with the recent report by Cowan et al demonstrating a reduction of AECOPD with IG treatment [9]. Conversely, in this case series, we have obtained a diagnostic immune evaluation, and initiated treatment with IG replacement or prophylactic antibiotics for patients who received a diagnosis of a humoral immunodeficiency. This case series provides a diagnostic approach to identify patients with immunodeficiency, it is a larger cohort than Cowan et al [9], and demonstrates that treatment of patient with IG or rotating antibiotics reduces frequency and severity of AECOPD. While we cannot recommend testing for immunodeficiency in patients with AECOPD, it seems reasonable to consider this differential diagnosis in those patients who have frequent and severe exacerbations despite maximal pulmonary therapy.
